# How Does a Port Build Influence? Diffusion Patterns in Global Oil Transportation

**DOI:** 10.3390/s22228595

**Published:** 2022-11-08

**Authors:** Peng Peng, Christophe Claramunt, Shifen Cheng, Feng Lu

**Affiliations:** 1 State Key Laboratory of Resources and Environmental Information System, Institute of Geographic Sciences and Natural Resources Research, Chinese Academy of Sciences, Beijing 100101, China; 2 Naval Academy Research Institute, Brest Naval, CEDEX 09, 29240 Brest, France

**Keywords:** global oil transportation, ports, vessel trajectory data, direct influence diffusion, indirect influence diffusion

## Abstract

Ports play a critical role in the global oil trade market, and those with significant influence have an implicit advantage in global oil transportation. In order to offer a thorough understanding of port influences, the research presented in this paper analyzes the evolution of the dominance mechanisms underlying port influence diffusion. Our study introduces a port influence diffusion model to outline global oil transport patterns. It examines the direct and indirect influence of ports using worldwide vessel trajectory data from 2009 to 2016. Port influences are modelled via diffusion patterns and the resulting ports influenced. The results of the case study applied to specific ports show different patterns and influence evolutions. Four main port influence trends are identified. The first one is that ports that have a strong direct influence over their neighboring ports materialize a directly influenced area. Second, geographical distance still plays an important role in the whole port influence patterns. Third, it clearly appears that, the higher the number of directly influenced ports, the higher the probability of having an influence pattern, as revealed by the diffusion process. The peculiarity of this approach is that, in contrast to previous studies, global maritime trade is analyzed in terms of direct and indirect influences and according to oil trade flows.

## 1. Introduction

Maritime transportation, in which ports play a critical role, is considered to be a major global trade mode. About 80% of global trade volume and more than 70% of global trade value are transported by maritime transportation and are handled by ports worldwide [1]. By combining better coordination between port terminals and liner shipping companies [2] and location advantages, it appears that several ports have achieved substantial influence on maritime transportation and potentially better integration of environmental issues [3]. For a port, influence is manifested by the port’s ability to dominate a certain amount of trade volume of ports that are directly or indirectly connected to it. Therefore, the greater the influence of the port, the more critical a role it plays in global trade. Moreover, the continuously extending maritime transportation network has been gradually concentrating around these ports, and the growing traffic distribution has also exhibited place-dependent characteristics [4]. Therefore, the emergence of port influence has generated the Matthew effect [5], which means that the influence of these significant ports continuously increases. In particular, in order to improve maritime transportation efficiency, shipping companies are very likely to prioritize these ports when designing a maritime trade route [6]. This leads to a few ports having a significant influence on other ports through cargo transportation. While these hub ports are generally well known, there is still a need to provide better modeling and understanding of the influence of these ports towards their maritime environments, dependent ports at different levels in the maritime network, and how such processes evolve and diffuse over time. Studying the formation mechanisms of the port influence, as well as identifying a modelling framework to do so effectively, should provide theoretical support to port decision-makers for optimization of trade relations and help to highlight the importance of ports in global trade.

The concept of influence diffusion originates from social networks [7]. A social network is a diffusion medium for ideas or innovations; whether an individual adopts an idea or innovation will be largely dependent on the decisions of the individual’s peers or neighbors in the social network [8]. For a port, transportation networks are an important medium for diffusing information throughout port influence networks. They act as the conduits by which the innovative efforts of port technology and oil companies are widely spread. Information spillover and externalities from the concentration of oil and ship companies in a port can influence the transportation network [9]. Conversely, the spread of a port’s influence can reinforce its influence, which, finally, can attract additional transportation routes and oil vessels.

Changes in the transportation volumes between ports will not only influence the ports with which they have direct trade relations but also influence the entire supply chain through transshipment relations, etc., and then indirectly influence these ports. Therefore, we must not only consider the direct influence of these ports but also the indirect influence of these ports in the transportation process when modelling the whole port influence and its diffusion process. In order to materialize this difference, we subdivide the concept of port influence into direct and indirect influences. Intuitively, we define a port’s direct influence as its ability to dominate a certain amount of trade volume of ports that have direct trade relations with that port and a port’s indirect influence as its ability to dominate a certain amount of trade volume of ports that are in relation with that port through other ports. This paper introduces a port influence diffusion model that comprehensively considers both the direct and indirect effects of a port influence diffusion process.

Oil is the “blood” of modern industry and an indispensable strategic resource for the survival and development of a country. It plays an inestimable role in safeguarding national economic, social development, and national defense security. Global oil trade continues to show tremendous growth [10] and is still a major concern for national and international policies. We introduce a structural and quantitative approach whose objective is to offer a global picture and understanding of global oil trade patterns, and especially dominance and port influence factors. The whole approach is experimented in the context of a case study of several ports using worldwide vessel trajectory data from 2009 to 2016. Using our port influence diffusion model, the objective is to assess the evolutionary development of port influence and study the main patterns of change at the global level. We believe that the results can provide a clearer understanding of global oil trade patterns and the respective roles of the influencing and influenced ports within a large maritime transportation network. The remainder of the paper is organized as follows. Section 2 briefly surveys related work and provides the research background and motivation of our research. The data and suggested model are introduced in Section 3, while the results of the case study are described in Section 4. Finally, Section 5 concludes the paper and outlines further work.

## 2. Literature Review, Research Background, and Motivation

A series of studies have applied network centrality measures, such as degree and betweenness centrality, to model port influence [11,12,13,14,15,16,17,18,19]. For instance, Kaluza, Kölzsch, Gastner, and Blasius [11] argued that Shanghai emerged as an important port in the maritime network because it was widely visited by ocean vessels, while Antwerp gained its stature through strong connectivity with other ports. Ducruet and Notteboom [12] confirmed that Singapore was the most vital port in 1996 and 2006 measured by betweenness centrality. By evaluating the robustness of different types of transport networks, Peng, Cheng, Chen, Liao, Wu, Liu, and Lu [15] showed that only a few key ports were of significant influence on the structural properties of the maritime transport network. As transshipment is an important pattern in cargo transportation, port influences might reveal some recursive properties. In other words, a significant port might not only influence ports directly connected to it but also ports not directly connected to it but in close relation through transshipments. Although the above-mentioned research generally reflects port influence patterns to a certain extent, as revealed by some network structural properties, the concept of port influence has multiple diffusion characteristics and cumulative effects that cannot be evaluated by network centralities alone. Integrating these successive trends is essential to intimately reflect the characteristics of good gradual flows between ports. In fact, and for instance, while some ports might have high degree values due to active trade relations with other ports, their influence on the maritime transportation network might be relatively weak. Therefore, a question that still arises is to make a clear difference between ports that have weak indirect influence with ones that have significant indirect influence.

Recently, the study of influence diffusion has been mainly addressed from social network research [7,20,21,22,23], including areas such as marketing [8,24,25], rumor propagation [26,27,28], and online advertisements [29,30,31]. A key issue that has been widely studied in influence diffusion is the maximum influence [32,33,34], which means finding the minimum number of nodes under a specific network diffusion model to maximize the influence. Kempe, Kleinberg, and Tardos [7] proposed two basic models to study propagation patterns in a social network, namely the independent cascade (IC) model and the linear threshold (LT) model. Lu et al. [35] proposed a comparative independent cascade (Com-IC) model by considering the full spectrum of entity interactions from competitive to complementary. The results show that the proposed algorithms consistently outperform intuitive baselines in four real social networks, namely Douban-Book, Douban-Movie, Flixster, and Last.fm. Hosseinpour et al. [36] designed an algorithm that considers both network and spatial properties to extend the influence maximization problem to location-based social networks; the results illustrated the performance of the proposed method in determining influential nodes that maximized socio-spatial influence.

The above-mentioned research provides a good reference for measurement of port influence. The focus of this paper is to search for the influence formation mechanism of global oil transportation ports by discovering the influence diffusion characteristics of different types of ports. This implies considering and modeling the influence diffusion characteristics of each port. While current research on maximizing influence from social networks provides some valuable analysis, it is not completely appropriate for studying port influence mechanisms and properties as most if not all these approaches are mainly structural and do not completely take into account maritime network traffic and direct and indirect dependence relationships. When studying port influence diffusion processes, Peng, Poon, Yang, Lu, and Cheng [9] introduced in a previous work an influence diffusion model to study the diffusion patterns of global oil ports. This study showed that Rotterdam, Antwerp, and Singapore appear as the three most influential ports, particularly in 2013 and 2016, and can influence ports in the entire network, while over half of the ports in the networks were able to influence just a single port. However, the way a port builds its influence network is not yet completely known. Moreover, it has been shown that the vast majority of oil ports belong to influenced ports, and the vast majority of these ports belong to regional ports [37], and, therefore, the respective influence of these ports is likely to vary.

In order to consider the respective influence of direct and indirect port relationships, the main objective of this paper is that, from global oil trade freights approximated from AIS data trajectories, a global maritime network can be constructed and its structural and diffusion properties further studied. We introduce a modelling approach whose objectives are to develop (1) a recursive analysis of port hub direct and indirect influences in the overall maritime network according to the oil trade case and (2) a temporal analysis to mine the formation mechanism of hub port influence. While our previous research was oriented to the study of port influence diffusion paths and evolution [9], a distinction was not made between direct and indirect influences, as well as the roles played by intermediate ports in these influences. In fact, as described in Section 4, this study emphasizes the significance of these two factors in determining a port’s influence. Overall, this study’s objective is to primarily focus on these two aspects to deeply mine the influence formation mechanism of port hubs.

## 3. Data and Modelling Approach

### 3.1. Data for Global Oil Transport Network

The trade relationship between ports is mainly realized by oil vessels. In order to infer a global oil transport network, vessel trajectories generated from AIS data from 2009 to 2016, and that capture vessel movements between ports, were considered [38,39,40]. AIS data and port attributes were collected from HiFleet Co., Ltd. (http://www.hifleet.com/) (accessed on 1 January 2009), an authoritative maritime data provider in Shanghai, China. AIS messages are automatically broadcasted with a reporting frequency, and the AIS data included both static information (name, type, length, and breadth, etc., of vessel) and dynamic information (real-time position, speed, course, heading, loading status, destination port, etc.) [41]. Table 1 provides some attributes closely related to calculation of arrival and departure records of vessels, including the Maritime Mobile Service Identity (MMSI) number (an identification number unique to each vessel) [42] and real-time positional information (port attribute data mainly include port name and location). AIS data and port attribute data support derivation of arrival and departure times of oil vessels at all calling ports and then generation of a reference dataset that comprises the journeys of all oil vessels during that period.

In this study, a weighted directed graph $G\;\left(N_{y},E_{y},W_{y}\right)$ is used to represent the annual global oil transportation network in this time period, with $N_{y}$ being the set of all nodes in *G* representing ports involved in oil trade in year y (2009,…, 2016); E denotes the set of all edges linking pairs of port nodes in $N_{y}$; and $W_{y}$ represents the weights for all trade routes, expressed by the total freight trade volume on each route in year y. Since the actual freight volume of each voyage cannot be accurately calculated, we retain the deadweight of each vessel to represent the vessel’s transport capacity. The freight volume of all journeys of a route is aggregated to represent each route weight.

### 3.2. Port Influence Diffusion Model

**Figure 1:**
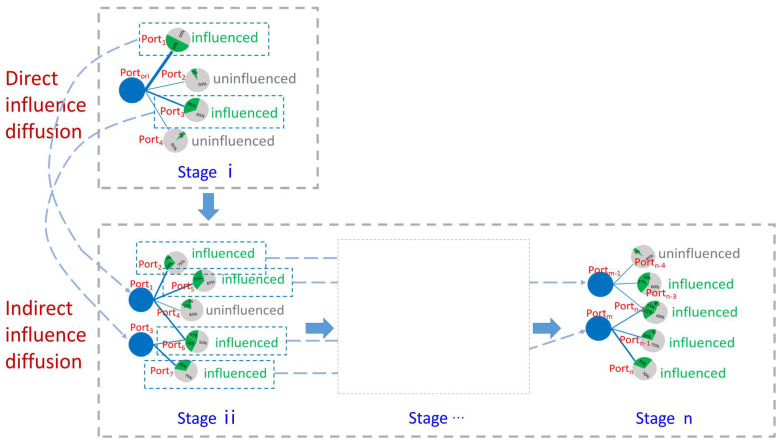
Schematic diagram of port influence diffusion model.

Figure 1 shows a schematic diagram of the port influence diffusion model. The diffusion model is recursively represented throughout successive diffusion “stages”. The first diffusion stage of a given port denotes the direct port influence diffusion by oil trade, which is materialized by its directly connected ports. The second indirect influence diffusion stage is given by the series of ports in relation by oil trade to the directly influenced ports.

As an influenced port’s influence diffuses over the entire network, other uninfluenced ports may turn “influenced” (labeled “influenced” around the port node) or may stay “uninfluenced” (labeled “uninfluenced” around the port node) during the diffusion stage. This pattern is modelled by the following Formula (1):

(1) $$f_{influence_{i}}(v)=\left\{\begin{array}{l} influenced\;\;\;\;\;\;\;\;\;\;\;\;\;\;\;\;\;\;\theta_{v}^{i}\geq \theta_{v}\;\;\\ uninfluenced\;\;\;\;\;\;\;\;\;\;\;\;\;\;\theta_{v}^{i}<\theta_{v} \end{array}\right.$$

(2) $$\theta_{v}^{i}=\sum_{u\in N{\left(v\right)}^{i}}b_{uv}^{i}$$

where $f_{influence_{i}}\left(v\right)$ models whether port v is influenced at the ith diffusion ($\;i=\left(1,2,\ldots \right)$). $\theta_{v}^{i}$ indicates the cumulative value of port v in the ith diffusion stage (Formula (2)). $\theta_{v}$ represents the threshold of changing into influenced status by its adjacent port nodes. When the accumulated influence of port v is equal to or greater than $\theta_{v}$, port v changes into an influenced status at the ith +1 diffusion stage and maintains the status until all the diffusion stages end. Otherwise, the port status is not changed, while the influence coefficient is accumulated at this diffusion stage. Without loss of generality, we set for that study the threshold value $\theta_{v}=0.2$ for all ports, which means that the port node is influenced when its cumulative influence (namely the freight volume) reaches at least 20% of its total influence (namely the total freight volume). It appears that this value is substantial enough to stand for a significant influence of the original diffusion port on a target diffusion port.

Stage *i* in Figure 1 represents the first influence diffusion stage. Here, $Port_{ori}$ stands for the initial influence diffusion port; the blue line represents the influence diffusion path, and the thickness captures the freight volume from the original diffusion port to the target diffusion port. Moreover, the green sector represents the proportion of the freight volume from $Port_{ori}$ to the target influenced port out of all the import freight volume to the target influenced port. Therefore, the influence coefficient value $b_{uv}^{i}$ of port u on port v at the ith diffusion ($i=\left(1,2,\ldots \right)$) is defined as follows:

(3) $$b_{uv}^{i}=\frac{FreightVolume_{u}^{out}(v)}{\sum_{w\in N{\left(v\right)}^{i}}FreightVolume_{v}^{in}(w)}$$

where $FreightVolume_{v}^{in}\left(w\right)$ represents all the freight volume from port w to port v (Formula (4)), and $FreightVolume_{u}^{out}\left(v\right)$ denotes all the freight volume from port u to port v (Formula (5)). $N{\left(v\right)}^{i}$ denotes all ports that transport cargo directly from other ports to port v at the ith diffusion.

(4) $$FreightVolume_{v}^{in}(w)=\sum VesselFreightVolume_{v}^{in}(w)$$

(5) $$FreightVolume_{u}^{out}(v)=\sum VesselFreightVolume_{u}^{out}(v)$$

where $VesselFreightVolume_{u}^{out}\left(v\right)$ represents the vessel freight volume departing from port u to port v. $VesselFreightVolume_{v}^{in}\left(w\right)$ denotes the vessel freight volume from port w to port v. The vessel deadweight approximates the freight volume.

As shown in Figure 1, port_1_ and port_3_ have an influenced status at the first diffusion stage, while other ports keep their status as uninfluenced. The influence in the first diffusion stage is defined as the direct influence of the initial diffusion port, and the direct influence ratio $r_{direct}$ is defined as in Formula (6):

(6) $$r_{direct}=\frac{n_{influence_{i}}\;}{k_{ori}^{out}}$$

$n_{influence_{i}}$ stands for the number of directly influenced ports by the initial influence diffusion port, and $k_{ori}^{out}$ represents the out degree of this initial influence diffusion port. The higher the value of $r_{direct}$, the greater the direct influence of the initial influence diffusion port.

Stage ii shows the second influence diffusion stage, and we define the diffusion stage after stage ii indirect influence diffusion. As shown in Figure 1, ports that have been changed into influenced status at the first diffusion stage are considered as diffusion ports at this stage. Moreover, the influence coefficient value at this diffusion stage can be set using Formulas (2) and (3) in order to derive the cumulative influence coefficient value of all ports at this stage.

For each port v, the accumulated influence value of all ports is bounded by the unit value as follows:

(7) $$\sum_{u\in N{\left(v\right)}^{i}}b_{uv}^{i}\leq 1$$

According to the above process, the influence diffusion stages for all ports in the network can be iteratively derived. Stage *n* shows the *n*th influence diffusion stage, namely the last effective influence diffusion to calculate all ports influenced by the initial port. It should be noted that, the higher the direct diffusion stage, the higher the number of transshipments from the original diffusion port to the influenced ports. More transport transshipment times will result in longer transport distance or transport time, which, in fact, denotes a lower efficiency of the port influence diffusion.

## 4. Modelling Application to the Port Case Study

**Figure 2:**
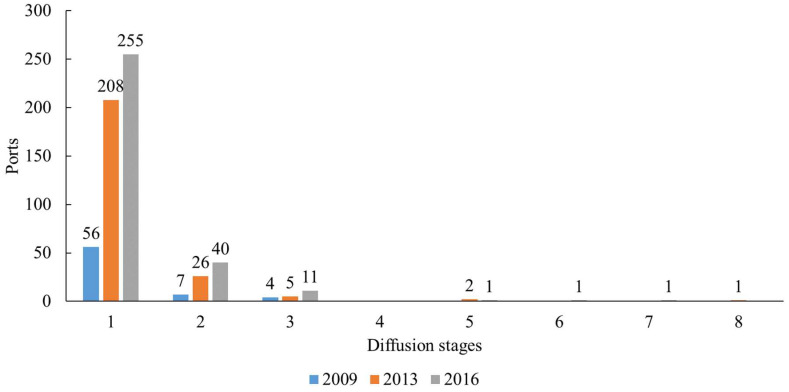
Number of influencing ports at different diffusion stages.

The modelling approach has been applied to study the evolution of the influence of all ports involved in the oil trade worldwide from 2009 to 2016. By visualizing the paths of influence diffusion at different stages, the previous work showed that diffusion of most port influences occurred at the first diffusion stage (Peng, Poon, Yang, Lu, and Cheng [9]). This principle is also retained at the first diffusion stage of our model, that is, to evaluate the direct influence of the ports. The main objective is to explore the formation mechanism of port influence by combining changes in the original diffusion port’s route, trade volume, and the geographical distribution of the influenced ports. We select 2009, 2013, and 2016 as representative years and visualize a typical port’s first influence diffusion stage to analyze the evolution of the port influence diffusion process. Amongst them, a total of 422 ports had an influence in 2009, 2013, and 2016, of which 67, 242, and 309 ports are influenced each year, respectively. Figure 2 shows the overall figures of the number of ports at different diffusion stages. For example, one can remark that eleven ports had three diffusion stages in 2016. It can be found that, although the number of ports that mainly have a direct influence at the first diffusion stage accounts for the vast majority, a few more ports have an indirect influence at later diffusion stages.

Our objective is then to explore the formation mechanisms of port influence by studying the mechanisms of the diffusion of ports’ routes and trade volumes as applied to global oil patterns, as well as the geographical distribution of the influenced ports. Through the analysis of the long-term port influence diffusion from 2009 to 2016, four main port influence evolutionary trends appear. The first one is where the port has a strong influence and shows continuous growth (type 1); the second is where the port’s influence remains relatively stable over time (type 2); the third is where the port’s influence shows a phased change, meaning that the port’s influence increases at first and then decreases (type 3); and the fourth is where, initially, the port’s influence is small but shows rapid growth (type 4).

**Figure 3:**
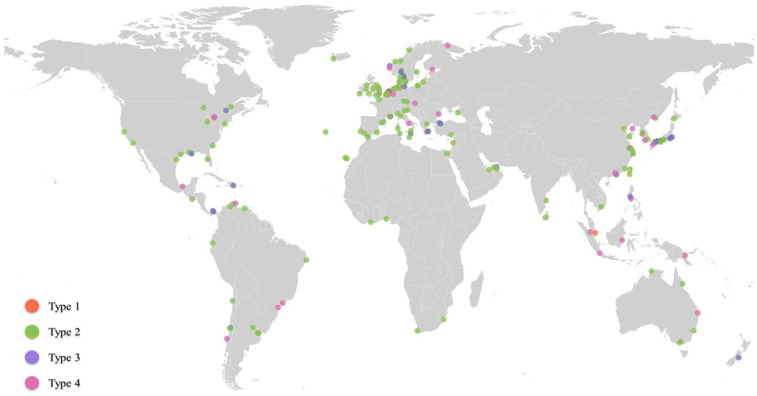
Geographical distribution of different types of ports.

Figure 3 shows the geographical distribution of these different types of ports. Amongst them, the ports belonging to type 1 are Rotterdam, Antwerp, and Singapore. Type 2 has the largest number of ports (i.e., 120); most of the world’s oil trading ports have maintained relatively stable influence diffusion characteristics, and the spatial distribution of the ports almost covers the areas involved in global oil trade. Ports with this diffusion characteristic are mainly located in countries with relatively long development time of oil trade as well as large demand for oil trade. These ports are mainly responsible for oil imports from the source country to the important ports of their own countries and undertake the function of transshipment to other ports in their own countries or neighboring countries. Type 3 ports (i.e., 28) are mainly located in key straits or countries and regions with large oil production and consumption needs, such as ports in Japan. This characteristic is caused by the change in influence of neighboring ports. Type 4 ports (i.e., 30) are mainly located in areas where oil trade has shown continuous growth, such as ports in East Asia, growing trade demand being the main reason for this trend. Almost all these ports did not involve oil trade in 2013.

### 4.1. Ports with Significant Influence and Continuous Growth

**Figure 4:**
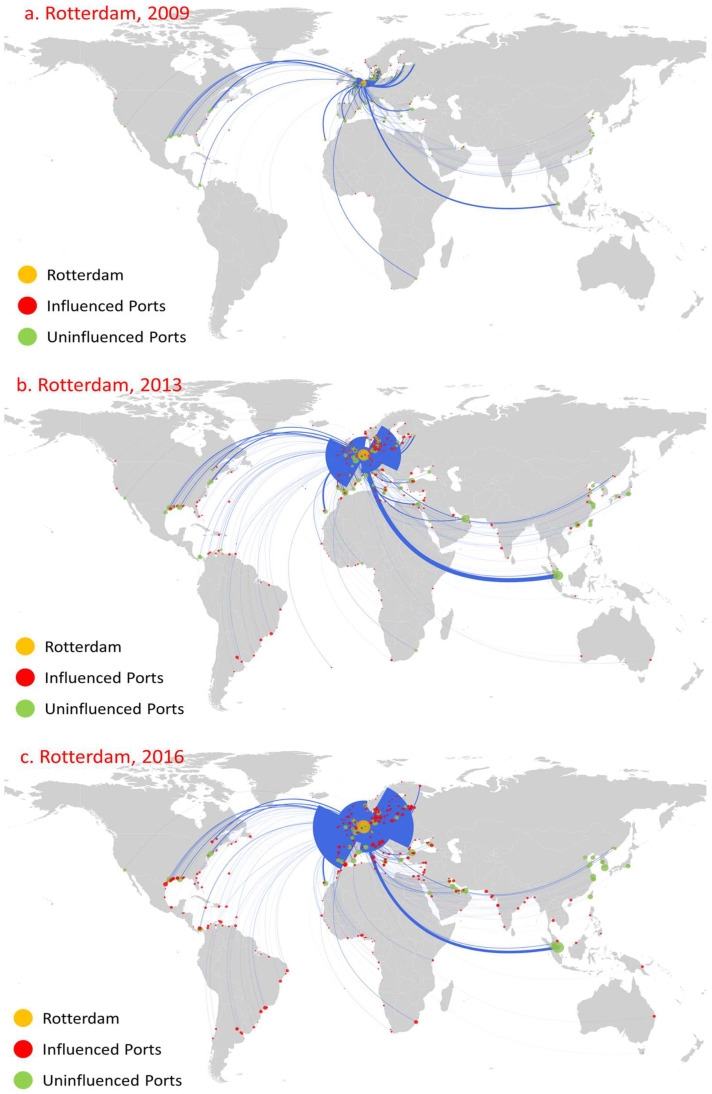
Influence diffusion at the first stage: Rotterdam. Note: the orange node denotes the initial diffusion port, while the blue line denotes the trade route from the initial diffusion port to the destination ports worldwide. The red node denotes the influenced node at the first stage, namely the directly influenced ports, while the green node denotes the uninfluenced node. (The same for the following Figure 5, Figure 6, Figure 8, Figure 9, Figure 10, Figure 11, Figure 12 and Figure 13).

**Figure 5:**
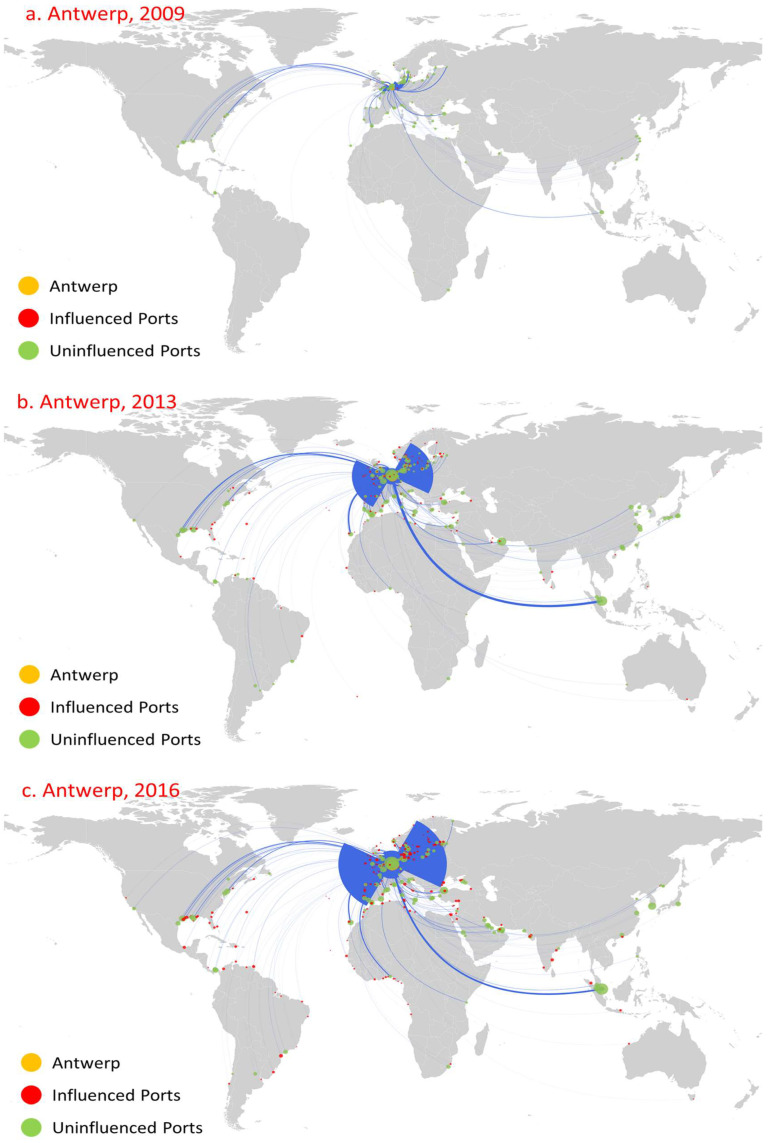
Influence diffusion at the first stage: Antwerp.

**Figure 6:**
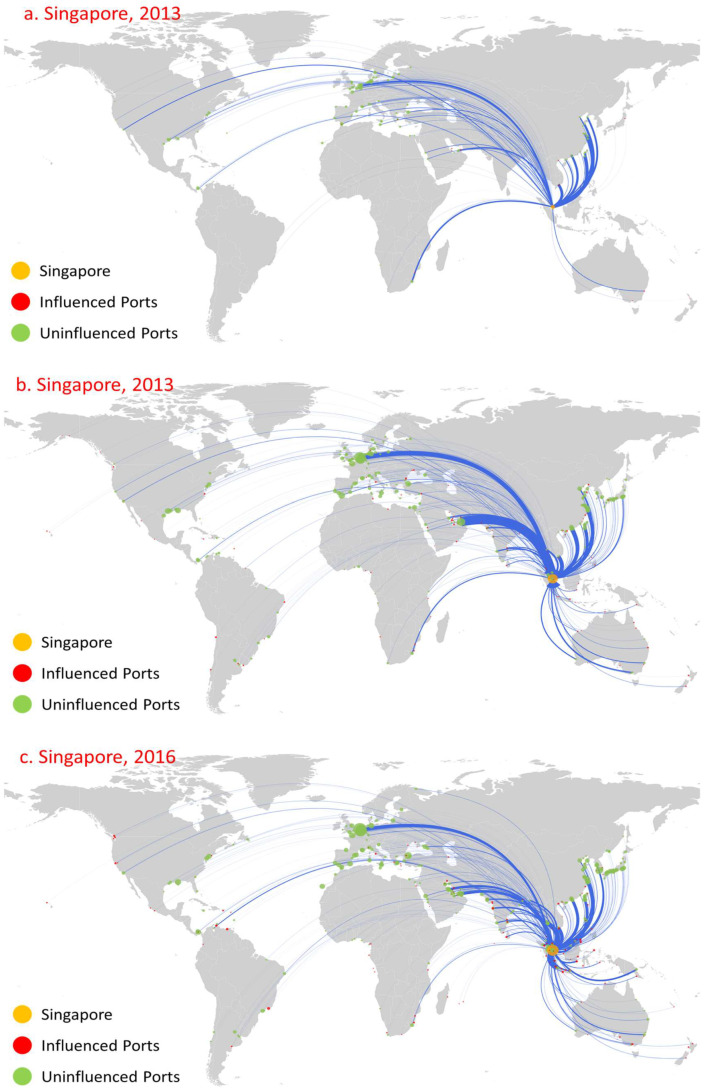
Influence diffusion at the first stage: Singapore.

Among the oil trade ports, Rotterdam, Antwerp, and Singapore are the top influential ports during the period considered and show significant growth trends. Overall, the influence of these three ports is significantly greater than that of all other ports, especially in 2013 and 2016. Figure 4, Figure 5 and Figure 6 show the first influence diffusion stage (namely direct influence) of Rotterdam, Antwerp, and Singapore, respectively. The geographical location of these three ports is extremely more beneficial, and the oil trade has developed for a long time. Meanwhile, with the continuous support of the government, there are advantages in perfect customs facilities and preferential tax policies [43,44,45], attracting Shell (Shell), BP, Exxon Mobil, ESSO, Gulf Oil, and other world multinational oil monopolies to build oil refining bases. Moreover, these three ports are also international shipping hubs and international trade centers [46]. Therefore, under the combined effect of various factors, they have formed significantly higher port influence than other ports.

Rotterdam began to form in 1620. The rise of the petrochemical industry has promoted rapid development of the port, and it has become an important cargo distribution center in Europe [47]. In 2009, the influence of Rotterdam was greater than other oil ports: there were 84 influenced ports, and its influenced areas were mainly concentrated in northwest Europe, the Mediterranean, and North America (Figure 4a). In stark contrast, although the number of routes is not far from Rotterdam, the direct influence of Antwerp (Figure 5a) and Singapore (Figure 6a) was relatively small: they only influenced 12 ports and 14 ports, respectively, and the distribution of these ports was relatively limited to their neighboring area. The indirect influence of the three ports is all relatively limited. For example, Antwerp had no indirect influence.

**Figure 7:**
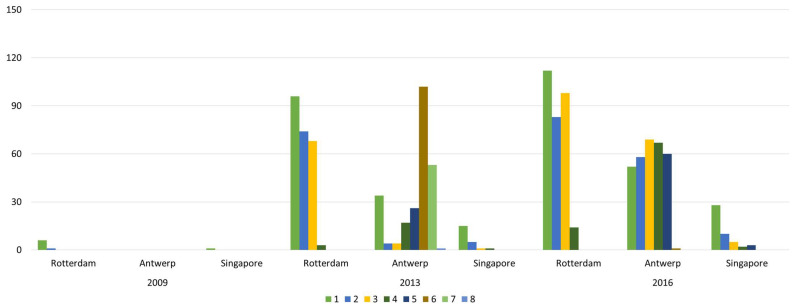
Number of influenced ports of influence at different diffusion stages: Rotterdam, Antwerp, and Singapore.

The global oil consumption has shown a growing trend over time, especially in the Asia-Pacific region, northwestern Europe, and the Americas [48]. As the oil trading hub linking these trading regions, the number of routes and trade volume of Rotterdam, Antwerp, and Singapore have shown an obvious growth trend (Figure 4b, Figure 5b, Figure 6b). The rapid development of navigation technology and the wide use of large vessels resulted in an increase in long-distance transportation, this having a significant accelerating effect on the diffusion of port influence. By 2013, the direct influence of the three ports showed a significantly increased trend, mainly reflected in the following three aspects. First, the number of ports that directly influenced showed significant growth. The number of ports directly influenced by Rotterdam, Antwerp, and Singapore increased to 313, 160, and 115, respectively, and the direct influence ratio of the three ports separately reached 77.28% (Rotterdam’s out-degree of 405), 49.23% (Antwerp’s out-degree of 325), and 32.86% (Singapore’s out-degree of 350), indicating that most of the ports with their trade relations were under their direct influence. Second, the geographical distribution of these influenced ports has shown great expansion. The ports that are directly influenced by Rotterdam covered 71 countries, and even 12 East Asia ports, including Shenzhen Port (Figure 4b). Antwerp’s directly influenced ports expand to northwest Europe, the Mediterranean region, and the Americas (Figure 5b). Singapore’s directly influenced ports began to spread rapidly to East Asia, the Americas, and the Mediterranean region (Figure 6b). Third, the number of directly influenced ports that can influence other ports has also shown a significant increase. Figure 7 shows the number of ports that can influence other ports, that is, Rotterdam, Antwerp, and Singapore, at different stages (1, 2, …, 8 denote the different diffusion stages). The directly influenced ports of Rotterdam, Antwerp, and Singapore, respectively, denote 96, 34, and 15 influenced ports with additional influences (Figure 5). However, most of these ports only influence a few ports.

Another pattern that appears is that the direct influence of these three ports greatly enhances their respective indirect influences. Specifically, the number of indirectly influenced ports also shows a significant increase. These three ports control most of the oil ports, especially Rotterdam and Antwerp, which influence almost all the ports within eight diffusion stages. Meanwhile, Rotterdam’s indirect influence diffusion efficiency is significantly greater than that of Antwerp and Singapore. As shown in Figure 7, 74 and 68 ports are influenced by Rotterdam at the second and third diffusion stages, respectively. Notably, the world’s top 10 most influential ports, except Ichihara (a port in Japan, influenced at the third diffusion stage), were all influenced at the second diffusion stage. For Antwerp, except for Kiel, the world’s top 10 ports with significant influence were influenced at the fifth or sixth diffusion stages, and most were influenced at the sixth diffusion stage. The conclusion here is that most of the ports were influenced at the sixth and seventh diffusion stages, indicating that, although the number of influenced ports was relatively large, the diffusion efficiency of indirect influence was relatively low.

By 2016, the direct influence of the three ports still shows a relatively growing trend. The number of directly influenced ports increased to 391, 228, and 138, respectively, with a direct influence ratio reaching 82.84% (out-degree of 472), 61.13% (out-degree of 373), and 34.67% (out-degree of 398), respectively. Moreover, the number of directly influenced ports that can influence other ports increases to 112, 52, and 28, respectively. Meanwhile, the influence diffusion also shows two significant characteristics. On the one hand, although the number of influenced ports shows a continued growth trend, the geographical distribution of influenced ports shows a more concentrated distribution. For example, Rotterdam had increased its influence over neighboring ports, such as the ports in the US and the UK, which, respectively, increased from 34 and 17 (in 2013) to 42 and 28 (in 2016). However, there was a slight decrease in the influenced ports geographically far away from Rotterdam. For instance, there was only one influenced East Asia port (Figure 4c). On the other hand, there exists path dependence in the diffusion of port direct influence. Among the ports directly influenced by Rotterdam, Antwerp, and Singapore in 2016, 209, 86, and 39 were the same as those directly influenced by it in 2013.

In addition, the strong direct influence of the three ports improves their indirect influence to a certain extent and the diffusion efficiency of the port (i.e., the number of ports influenced at lower diffusion stages shows an increasing trend). Moreover, port diffusion efficiency can be greatly improved by strengthening port influence on ports with significant influence at a relatively lower diffusion stage. For example, among the world’s top 10 significant influential ports, Rotterdam, Amsterdam, Istanbul, and Balboa were all influenced by Antwerp during the third diffusion stage, and its diffusion efficiency is greatly improved. The most direct embodiment of this is the ports influenced by Antwerp’s first five diffusion stages, which amount to 91.80% of all influenced ports compared with 31.8% in 2013.

### 4.2. Ports Maintain a Stable Influence

**Figure 8:**
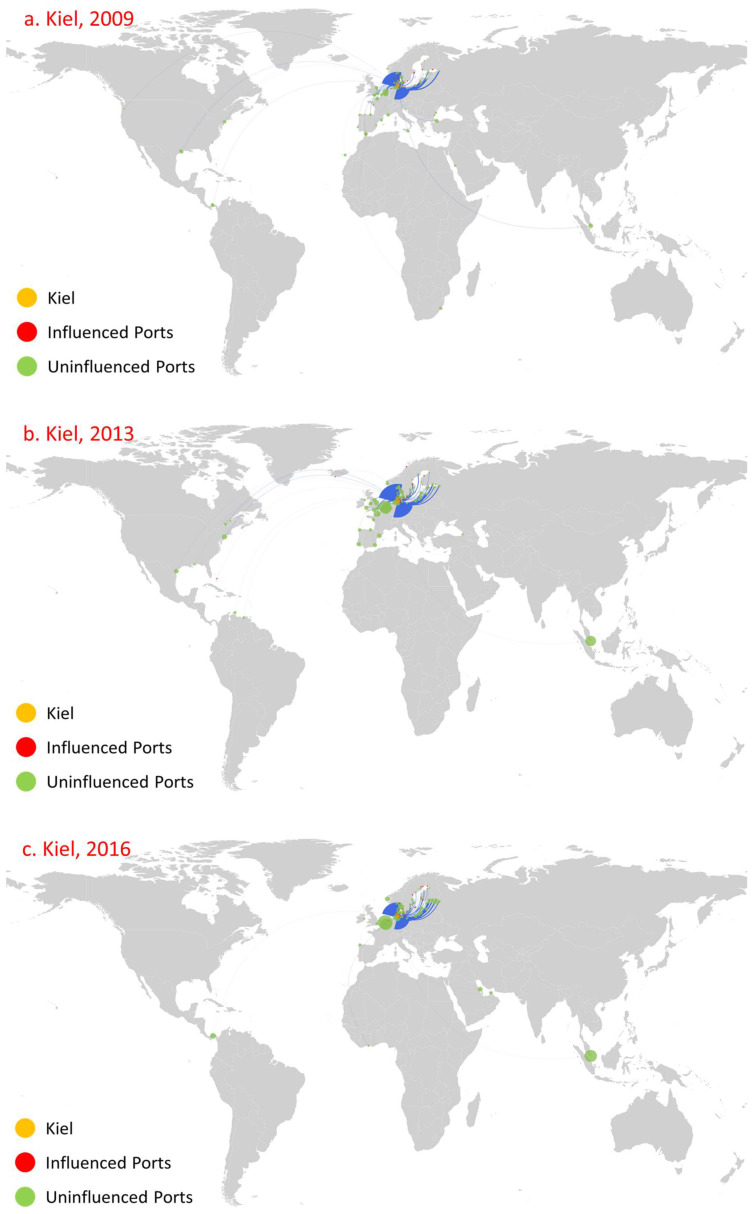
Influence diffusion at the first stage: Kiel.

**Figure 9:**
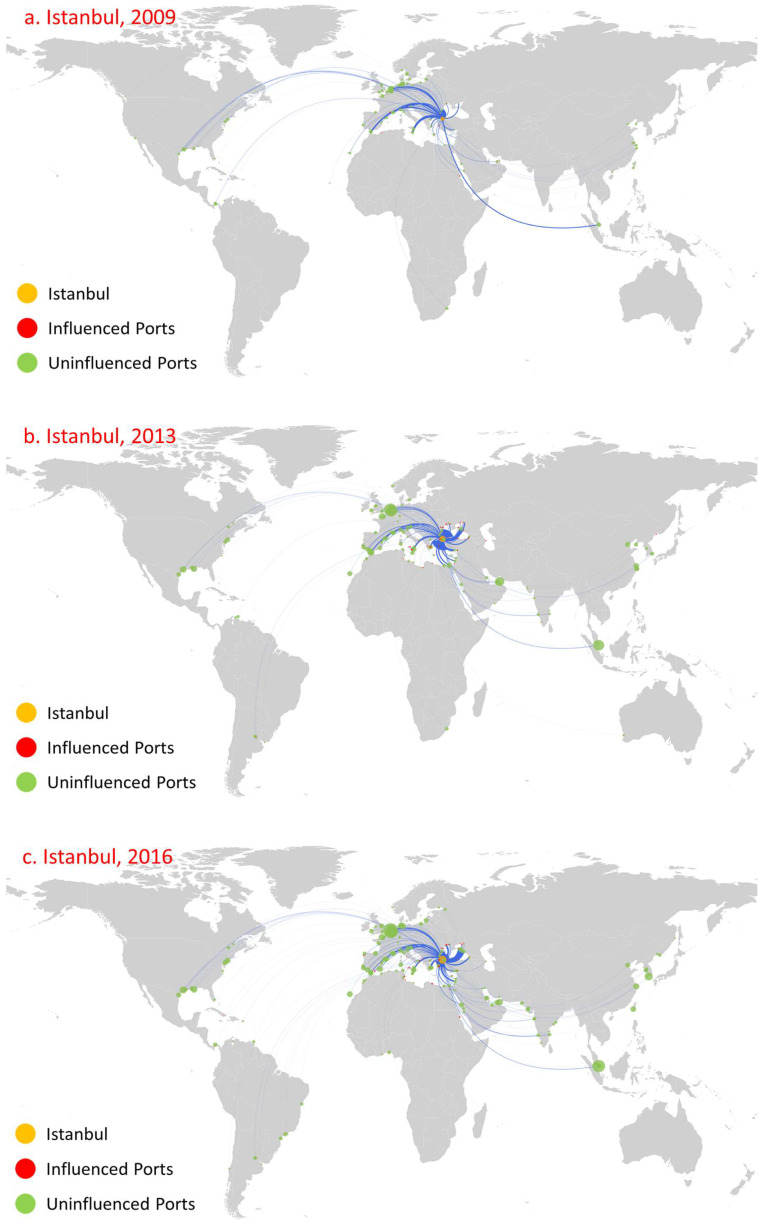
Influence diffusion at the first stage: Istanbul.

**Figure 10:**
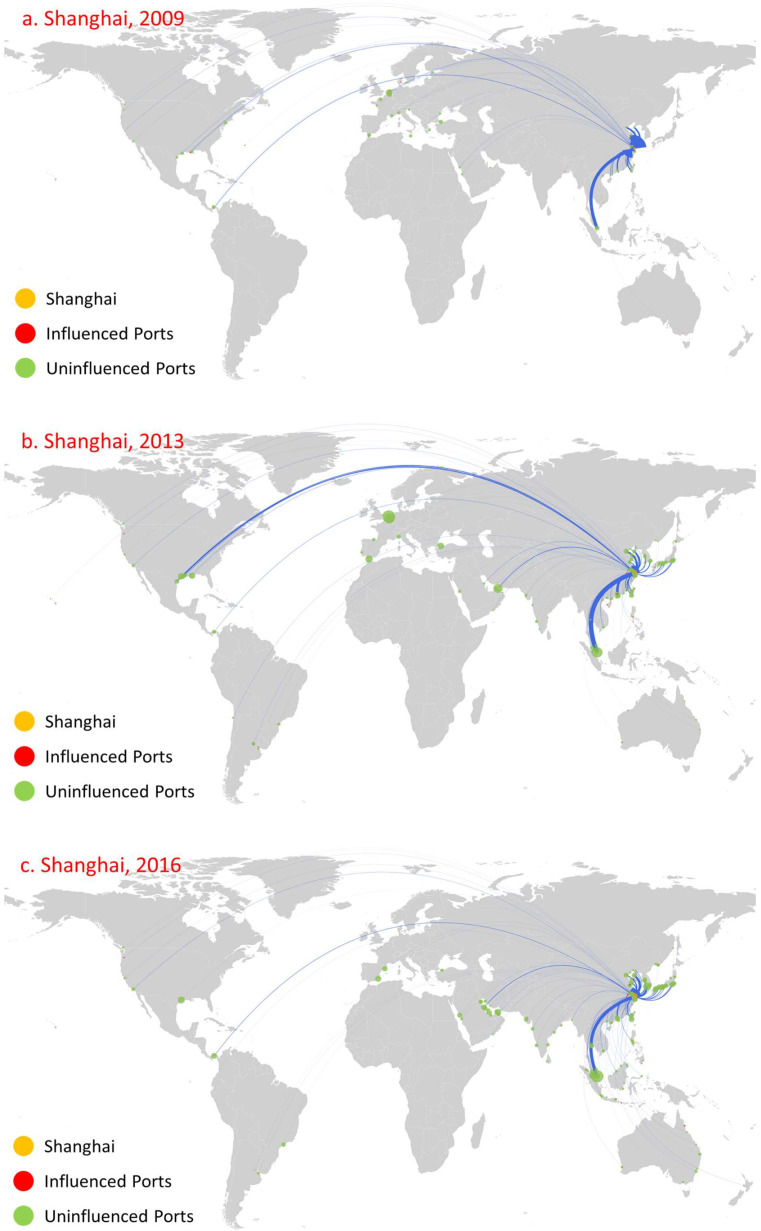
Influence diffusion at the first stage: Shanghai.

We select Kiel, Istanbul, and Shanghai as relevant cases for analysis (Figure 8, Figure 9 and Figure 10, respectively). The main reason behind this choice is that the countries to which these ports belong have huge demand for oil (Kiel in Germany, Shanghai in China; both are major oil-importing countries) and they serve as main oil transshipment hubs for their own countries, or just as a stable transshipment hub (Istanbul in Turkey controls the passage from the Mediterranean Sea through the Marmara Sea to the Black Sea). Moreover, these ports have been involved in oil transportation for a long time, as well as that they maintain relatively stable numbers of routes and trade volume, as shown in Figure 8, Figure 9 and Figure 10. Moreover, these ports have the following influence diffusion characteristics. First, they maintained a relatively stable influence in terms of the number of ports over this time period. Kiel influenced 28, 33, and 32 ports in 2009, 2013, and 2016, respectively. Shanghai influenced eight, fifteen, and fifteen ports in 2009, 2013, and 2016, respectively. Istanbul shows a little difference. In 2009, the number of influenced ports was relatively small (11), while it influenced 47 and 46 ports in 2013 and 2016, respectively. Second, the direct influence of these ports is relatively large, with most ports influenced at the first diffusion stage. Meanwhile, these ports are mainly distributed in their neighborhood region. For Kiel, even in 2009, the minimum number of ports influenced at the first stage was 22 amongst 28 overall, and these ports were concentrated mainly in northwestern Europe, such as Flensburg in Sweden. For Istanbul, the number of ports influenced at the first diffusion stage reached 11 in 2019, 43 in 2013, and 41 in 2016, while the influenced ports were mainly concentrated in the Mediterranean and Black Sea regions. For Shanghai, the number of ports influenced at the first diffusion stage reached eight in 2019, fifteen in 2013, and thirteen in 2016, and they were mainly concentrated in China. Third, there is path dependence and a geographical distribution pattern that appears among the directly influenced ports for all years. For instance, for Kiel, Rendesburg, Rodby, Trelleborg, and Turku were influenced in 2009, 2013, and 2016. More significantly, there were 10 influenced ports in 2016 in line with 2013, and the main reason is the huge consumer market of its home country. For Istanbul and Shanghai, eighteen and eight influenced ports constantly appear.

### 4.3. Ports with Phased Influence Change

**Figure 11:**
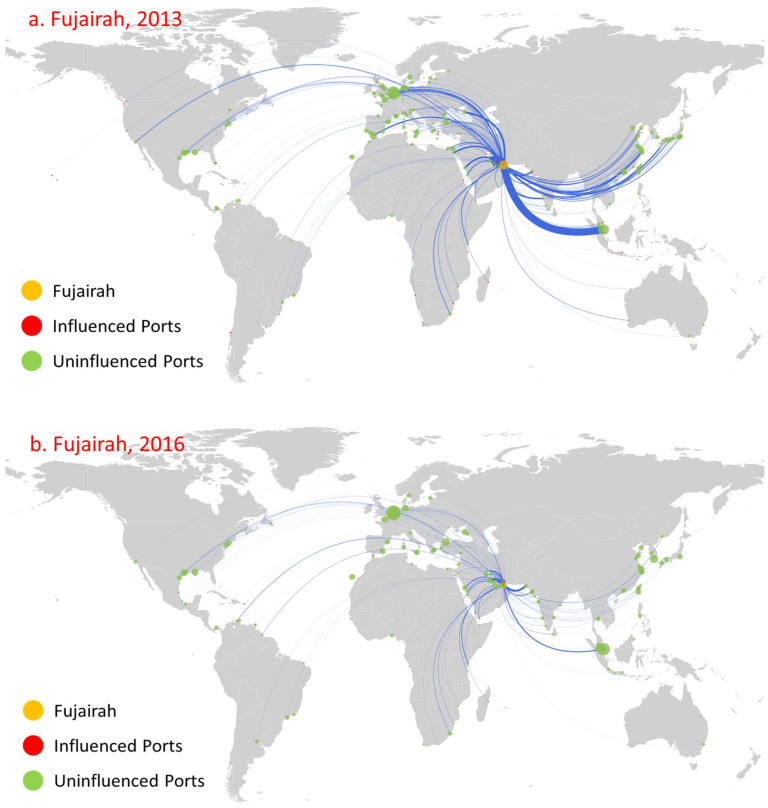
Influence diffusion at the first stage: Fujairah.

**Figure 12:**
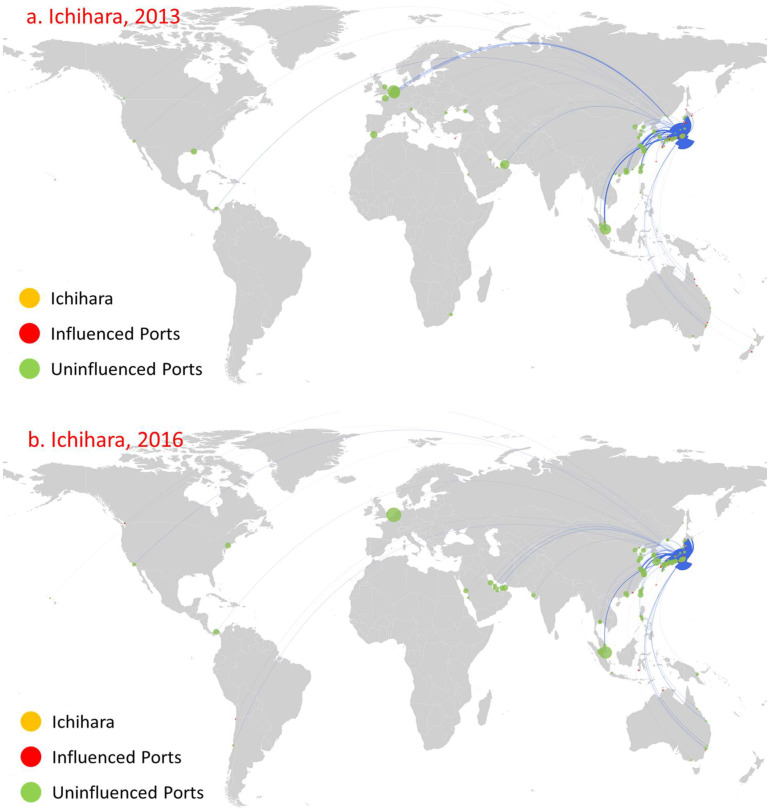
Influence diffusion at the first stage: Ichihara.

Figure 11 and Figure 12, respectively, show the diffusion of Fujairah in the UAE, and Ichihara in Japan. The main reason behind the trends that appear is that the competition of neighboring ports leads to a significant decrease in trade routes and trade volume (Figure 11 and Figure 12). First, the number of routes and trade volume showed a significant reduction trend. Meanwhile, the number of ports influenced by direct or indirect diffusions also showed a significant reduction. Moreover, the development of neighboring ports influences a port’s influence to a certain extent, which causes its influence to fluctuate. In 2009, both ports were not involved in the oil trade. By 2013, the number of directly influenced ports reached 54, which was less than those of Singapore, ranking fourth. By 2016, with further improvement in Singapore’s direct influence, the number of ports directly influenced fell by one to only 16. For Ichihara, it influenced 46 ports in 2013. By 2016, the influence of Yokohama increased, forming more obvious competition and leading to a decline in Ichihara’s influence, reducing the number of ports it influenced to 28. However, Ichihara presents some differences when compared with Fujairah. The influence decline of Ichihara mainly reflects a decrease from fifteen to three ports in its indirect influence, while it directly influenced 31 ports in 2013, mostly domestic ports. The number of ports directly influenced fell slightly to 25 in 2016. Second, the geographical distribution of ports directly influenced shows a significant contraction trend. For example, the geographical distribution of the directly influenced ports also shrank from being concentrated in the Middle East, the Red Sea, Southern Africa, and the Mediterranean to concentration only in the Middle East and the Red Sea. Third, there exists path dependence in the diffusion of port direct influence. Amongst the ports directly influenced by Fujairah and Ichihara in 2016, five and thirteen remain the same as in 2013, respectively.

### 4.4. Ports with Little Influence but Rapid Growth

**Figure 13:**
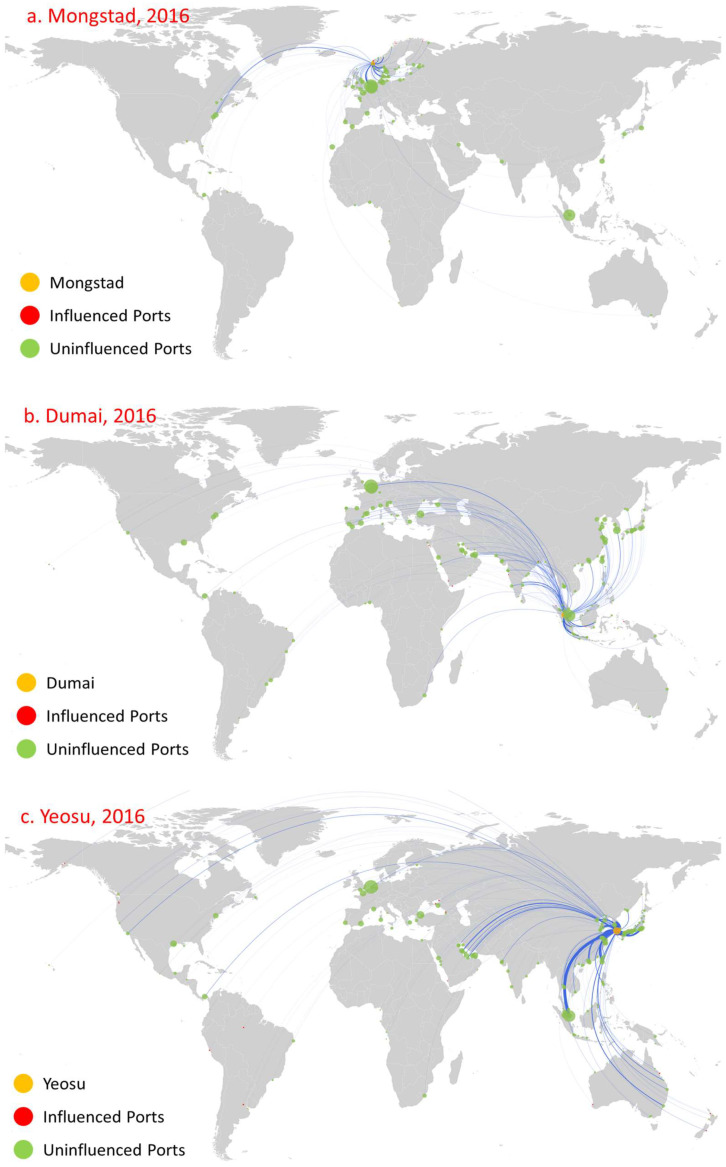
Influence diffusion at the first stage: Mongstad, Dumai, and Yeosu.

We selected northwest Europe (Mongstad in Norway, Figure 13a), the important straits (Dumai in Indonesia, Figure 13b), and East Asia (Yeosu in Korea, Figure 13c) for analysis. These ports mainly serve as small regional oil trade centers. With the increase in oil demand and oil production, their direct influence has improved and shows short distance diffusion character. However, due to a relatively short development period, their indirect influence has been limited. Yet, these ports have the potential to develop into significantly influential ports. For example, prior to 2013, only Dumai was involved in the oil trade and influenced only one port. By 2016, the number of ports influenced rose to seven, eleven, and twenty, respectively, for the three ports, and the geographical distribution of directly influenced ports was concentrated near them. These ports only had two diffusion stages. At the second diffusion stage, Yeosu influenced six ports, while the indirect influence of both Mongstad and Dumai was relatively weak, only influencing one port.

## 5. Discussion and Conclusions

The research presented in this paper introduced a port influence diffusion model to thoroughly analyze the evolution characteristics of the influence of global oil ports. The methodology considers two aspects: a port’s direct and indirect influence based on vessel trajectory data. The model identifies the influence of each port via its influence diffusion patterns and, recursively, the ports influenced in the network, this providing a sort of dominance maritime network model. The methodological novelty of the approach lies in the combination of an approximation of global oil freight patterns as derived from AIS trajectories and the application of a structural and quantitative analysis of the oil trade network that emerges amongst the different ports. The results that appear from our case study highlight four main trends of the port influence and dominance model: (1) ports that start off with and maintain a strong influence, reflecting a trend of continuous influence growth; (2) ports that have the same influence over time, thereby maintaining a relatively stable status; (3) ports that have influence that is phased; and (4) ports with initially little influence but with rapid growth.

The findings of our approach regarding the diffusion patterns that appear from global oil trade patterns can be summarized as follows. We found that ports that have a strong direct influence control their neighboring ports, thereby creating a direct influence range. Subsequently, the influenced ports show path-dependent characteristics, strongly correlated with geographical distance. By expanding the geographical scope and increasing the number of ports of influence via direct influence, indirect influence increased further. Moreover, port diffusion efficiency improved when there was a greater influence on ports at an earlier diffusion stage. Overall, this study analyzes the evolution of the influence of global oil transportation ports. As such, it provides a theoretical reference for evaluating the impact of port influence patterns and potentially support for further planning and mitigating actions, development of global oil trade regulations, as well as methodological support for analysis of the influence of other types of maritime ports (e.g., container ports).

When a port’s trade volume is influenced by external factors, such as freight rates, demand volume trends in various regional markets, and geopolitics, this not only affects the ports that trade with it directly but also indirectly influences ports by creating multiple stages of diffusion. As a result, the common order of global oil transportation is significantly affected through the diffusion of port influence, especially a port with great influence, creating additional risks in global oil trade. The peculiarity of our approach is that it not only highlights port direct influence patterns but also indirect influence on other ports that do not have direct trade relations with them. Therefore, this provides a rather comprehensive and extensive analysis of these influence patterns at the global level.

From our analysis, it appears that, among the global oil transportation ports, Rotterdam, Antwerp, and Singapore are far stronger than other ports based on both the number of ports directly and indirectly influenced and the geographical distribution of these ports, with their influence also showing continuous growth. However, some of the world’s major oil consumption regions (such as East Asia and the Americas) have not fared as well as the three major ports referenced here. According to our research results, we found that, although oil consumption in East Asia accounted for the majority of the global market, most ports in East Asia had less direct influence on other ports. Ports such as Shanghai in China and Yeosu in South Korea have the potential to develop into more influential ports, and an increase in these ports’ influence will reduce trade risks to some extent. However, it should be noted that the characteristics of tradability, strategy, and politics, as related to global oil trade, also show that oil issues cannot be purely economic or purely political. Therefore, two suggestions are proposed to improve a port’s influence. On the one hand, loose trade policies at the national level should help to increase a port’s influence. As mentioned above, Yeosu is a port with little influence but rapid growth, and the port’s rising influence status has partly been bolstered by the government’s loosening of regulations to allow international oil trading companies to blend and mix fuels [49]. On the other hand, learning from the influence formation mechanism of ports with great influence will also improve port influence. The underlying development mechanisms of the ports of Rotterdam, Antwerp, and Singapore included directly controlling neighboring ports, continuously increasing the number of ports under their influence, and expanding to influence ports farther away; by doing so, their direct influence increased. Meanwhile, these steps also promoted the indirect influence of the ports to a certain extent, and, by increasing their influence, the risk in global oil trade was reduced and trade stability enhanced to a certain extent.

There is still room for this research to be expanded. The recent pandemic and Russia–Ukraine conflict have shown the extreme volatility of maritime transportation trade flows, as well as their critical role in securing vital imports and exports. Clearly, this demonstrates that the role played by different ports in the maritime global network is time-dependent and may be influenced by these events. Thus, this is a strong incentive to broaden our research in different directions. First, there is a need to develop automated data acquisition processes to support real-time monitoring of the global maritime network; this is undoubtedly an expected development for stakeholders as well as policymakers. Second, the port influence model can be extended to investigate the causal factors that potentially explain port influence patterns. Third, additional trade volumes could be considered to provide a more balanced assessment of trade patterns. Fourth, additional quantitative network analysis at complementary spatial and temporal scales is necessary.

## Figures and Tables

**Table 1:** Some attributes of AIS data.

MMSI	Update Time	Longitude	Latitude
111111110	4 January 2016 21:03:49	118.6000	24.8333
111111110	13 January 2016 09:30:37	120.2333	31.9167
205073000	5 February 2016 08:19:50	56.3833	25.3500
···	···	···	···
